# Persisting alterations of iron homeostasis in COVID-19 are associated with non-resolving lung pathologies and poor patients’ performance: a prospective observational cohort study

**DOI:** 10.1186/s12931-020-01546-2

**Published:** 2020-10-21

**Authors:** Thomas Sonnweber, Anna Boehm, Sabina Sahanic, Alex Pizzini, Magdalena Aichner, Bettina Sonnweber, Katharina Kurz, Sabine Koppelstätter, David Haschka, Verena Petzer, Richard Hilbe, Markus Theurl, Daniela Lehner, Manfred Nairz, Bernhard Puchner, Anna Luger, Christoph Schwabl, Rosa Bellmann-Weiler, Ewald Wöll, Gerlig Widmann, Ivan Tancevski, Judith-Löffler-Ragg, Günter Weiss

**Affiliations:** 1 Department of Internal Medicine II, Medical University of Innsbruck, Anichstraße 35, 6020 Innsbruck, Austria; 2 Department of Internal Medicine, St. Vinzenz Hospital, Zams, Austria; 3 Department of Internal Medicine V, Medical University of Innsbruck, Innsbruck, Austria; 4 Department of Internal Medicine III, Medical University of Innsbruck, Innsbruck, Austria; 5 Clinic for Rehabilitation Münster and Karl Landsteiner Institut für Interdisziplinäre Forschung am Reha Zentrum Münster, Münster, Austria; 6 Department of Radiology, Medical University of Innsbruck, Innsbruck, Austria; 7 Christian Doppler Laboratory for Iron Metabolism and Anemia Research, Innsbruck, Austria

**Keywords:** COVID-19, SARS-CoV-2, Iron metabolism, Hyperferritinemia, Hepcidin

## Abstract

**Background:**

Severe coronavirus disease 2019 (COVID-19) is frequently associated with hyperinflammation and hyperferritinemia. The latter is related to increased mortality in COVID-19. Still, it is not clear if iron dysmetabolism is mechanistically linked to COVID-19 pathobiology.

**Methods:**

We herein present data from the ongoing prospective, multicentre, observational CovILD cohort study (ClinicalTrials.gov number, NCT04416100), which systematically follows up patients after COVID-19. 109 participants were evaluated 60 days after onset of first COVID-19 symptoms including clinical examination, chest computed tomography and laboratory testing.

**Results:**

We investigated subjects with mild to critical COVID-19, of which the majority received hospital treatment. 60 days after disease onset, 30% of subjects still presented with iron deficiency and 9% had anemia, mostly categorized as anemia of inflammation. Anemic patients had increased levels of inflammation markers such as interleukin-6 and C-reactive protein and survived a more severe course of COVID-19. Hyperferritinemia was still present in 38% of all individuals and was more frequent in subjects with preceding severe or critical COVID-19. Analysis of the mRNA expression of peripheral blood mononuclear cells demonstrated a correlation of increased ferritin and cytokine mRNA expression in these patients. Finally, persisting hyperferritinemia was significantly associated with severe lung pathologies in computed tomography scans and a decreased performance status as compared to patients without hyperferritinemia.

**Discussion:**

Alterations of iron homeostasis can persist for at least two months after the onset of COVID-19 and are closely associated with non-resolving lung pathologies and impaired physical performance. Determination of serum iron parameters may thus be a easy to access measure to monitor the resolution of COVID-19.

**Trial registration:**

ClinicalTrials.gov number: NCT04416100.

## Introduction

Two hallmarks of severe COVID-19 are hyperinflammation, most typically involving a “cytokine storm” with massive interleukin 6 (IL6) expression, and hyperferritinemia [1]. Ferritin is the most relevant cellular iron storage protein and is regulated by both, iron availability and inflammation [2, 3]. Accordingly, IL6 is a key mediator of inflammation-driven iron handling, as it induces the production of hepcidin, the master regulator of iron homeostasis [4]. Hepcidin regulates cellular iron efflux via degradation of the sole cellular iron exporter ferroportin 1 (FPN1), which induces cellular iron retention in macrophages and reduces duodenal iron absorption [5, 6]. Inflammation, therefore, causes alterations of iron homeostasis hallmarked by functional iron deficiency (ID) as reflected by high iron content in reticuloendothelial cells and consequently high serum ferritin levels whereas circulating iron levels are low. Subsequently, inflammation limits this metal’s availability for erythropoiesis, thus causing anemia, termed as anemia of inflammation (AI) [7]. AI is highly prevalent in patients with infections since the underlying immune-mediated iron restriction is considered as an important host defense mechanism to limit microbial proliferation and pathogenicity. Indeed, iron is not only essential for multiple cellular processes for eucaryotes but also for microbes including viruses [8–13]. Of importance, over 80% of hospitalized patients with COVID-19 presented with inflammation-driven imbalances of iron homeostasis upon admission, which predicted an adverse clinical course [14]. As ferritin also has pro-inflammatory properties, it has been speculated whether or not hyperferritinemia in COVID-19 might contribute to its pathogenesis and severity [15–17]. Accordingly, we herein analysed for persisting alterations of iron metabolism in survivors of COVID-19 aiming to evaluate their prevalence and their association with persisting pathologic processes linked to COVID-19.

## Methods

### Patients and study design

The development of interstitial lung disease (ILD) in patients with severe acute respiratory syndrome coronavirus 2 (SARS-CoV-2) infection (CovILD) study is an ongoing prospective multi-centre observational cohort trial aiming to systematically follow patients after COVID-19 (ClinicalTrials.gov number, NCT04416100). A total of 109 patients, aged 18 years or older, who previously suffered from mild to critical COVID-19 were included. All participants gave informed written consent and the study was approved by the local ethics committee at the Innsbruck Medical University (EK Nr: 1103/2020). The inclusion algorithm is depicted in Additional file 1 Fig. S1. Diagnosis of COVID-19 was based on typical clinical symptoms and a positive RT-PCR SARS-CoV-2 result obtained from a nasopharyngeal or oropharyngeal swab. Patients were evaluated 60 days (SD ± 12) after the onset of first COVID-19 symptoms, including clinical examination, medical history assessment, a structured questionnaire to assess typical COVID-19 symptoms, performance evaluation [e.g. six-minute walking test (SMWT)] and the acquisition of blood.

### Blood sampling and analysis

Blood samples were taken via routine peripheral vein puncture and analysed by standardized ISO-certified procedures. Additionally, peripheral blood mononuclear cells (PBMCs) were obtained via Ficoll–Paque separation (Pharmacia^®^, Uppsala, Sweden) from whole blood and EDTA or heparin blood was separated via centrifugation at 300×*g* to collect serum or plasma, respectively, as previously described in detail [18]. PBMC cell pellets were stored at − 80 °C until further use.

### RNA preparation and RT-PCR

We extracted total RNA from PBMC cell pellets using a guanidinium-isothiocyanate-phenol-chloroform-based protocol followed by reverse transcription of mRNA into cDNA, as detailed elsewhere [18]. TaqMan-PCR primers and probes or SYBR-Green primers were designed, and real-time PCR quantification was carried out with Bio-Rad^®^ CFX96 qPCR system using SsoAdvanced™ universal probes supermix (Bio-Rad Laboratories, Hercules, 152 CA). A list of the primers and probes sequences is depicted in Additional file 1 Table S1.

### Definition of anemia, iron deficiency and hyperferritinemia

Iron deficiency (ID) was assessed by ferritin, transferrin saturation (TSAT), soluble transferrin receptor and the soluble transferrin receptor/log ferritin index (sTFRF index), as previously described [19]. TSAT < 20% in combination with serum ferritin < 100 µg/L was defined as absolute ID, whereas a TSAT < 20% with serum ferritin > 100 µg/L was considered to reflect functional ID [20, 21].

Anemia was diagnosed according to hemoglobin (Hb) concentrations and gender, whereby a Hb below 120 g/L for women and a Hb below 130 g/L for men were used as cut-offs. The sTFRF index, TSAT and ferritin were used to differentiate between absolute and functional iron deficiency in the setting of anemia [21–23]. Accordingly, anemia was categorized as iron deficiency anemia (IDA, sTFRF index > 2, TSAT < 20%, serum ferritin < 30 µg/L), anemia of inflammation (AI, TSAT < 20% and serum ferritin > 100 µg/L or serum ferritin 30-100 µg/L and sTFRF index < 1), a combination of both (IDA + AI, TSAT < 20%, serum ferritin 30-100 µg/L, sTFRF index > 2) or unclassifiable anemia (TSAT normal or reduced, serum ferritin > 30 µg/L, sTFRF index 1–2), as previously described [24].

Hyperferritinemia was defined by a serum ferritin > 200 µg/L for women and > 300 µg/L for men, as previously reported [25].

### Analysis of lung involvement with computed tomography

60 days after COVID-19 onset, all study participants were evaluated with a low-dose (100 kVp tube potential) computed tomography (CT) scan of the chest. CT was acquired on a 128 slice multidetector CT hardware with a 38.4 × 0.6 mm collimation and spiral pitch factor of 1.1 (SOMATOM Definition Flash, Siemens Healthineers, Erlangen, Germany). CT images were evaluated for the presence of ground-glass opacities (GGO), consolidations, bronchiectasis, and reticulations as defined by the glossary of terms of the Fleischner society [26]. The severity of pathological pulmonary findings was graded for every lobe using the following severity score: 0—none, 1—minimal (subtle GGO, very few findings), 2—low (several GGO, subtle reticulation), 3—moderate (multiple GGO, reticulation, small consolidation), 4—marked (extensive GGO, consolidation, reticulation with distortion), and 5—massive (massive findings, parenchymal destructions). The maximum score was 25 (i.e. maximum score 5 per lobe).

### Statistical analysis

Statistical analyses were performed with statistical analysis software package (IBM SPSS Statistics version 24.0, IBM, USA). Descriptive statistics included tests for homoscedasticity and data distribution (Levene test, Kolmogorov–Smirnov test, Shapiro–Wilk test and density blot/histogram analysis). According to explorative data analysis, we used the following tests: Mann–Whitney *U* test and Kruskal–Wallis test for group comparisons of continuous data, Fisher’s exact test or Chi-square test for binary and categorical data and Spearman rank test to assess correlations. Multiple testing was adjusted by Sidak formula, as appropriate.

## Results

### Patient characteristics

Subjects were evaluated at a mean of 60 days (SD ± 12 days) after the onset of COVID-19 associated symptoms. The mean age was 58 years (SD ± 14 years) and the majority of participants were male (60%). Detailed characteristics of the cohort including a description of comorbidities are depicted in Table 1. According to the need of medical treatment, disease severity ranged from mild to critical: mild [outpatient treatment, N = 22 (20%)], moderate [inward treatment without respiratory support, N = 34 (31%)], severe [inward treatment with additional oxygen therapy, N = 35 (32%)], whereas 18 patients (17%) had critical disease with the need for mechanical ventilation at an intensive care unit (ICU).

**Table 1:** Demographics and clinical characteristics of patients enrolled in CovILD

	N = 109
Characteristics	
Mean age, years (SD)	58 (14)
Female sex, no. (%)	44 (40)
Median body mass index (SD)^a^	26.7 (4.8)
Comorbidities, no. (%)	
None	21 (19)
Cardiovascular disease	44 (40)
Hypertension	32 (29)
Pulmonary disease	21 (19)
Endocrine disease	49 (45)
Hypercholesterolemia	24 (22)
Diabetes mellitus, type 2	20 (18)
Chronic kidney disease	7 (6)
Chronic liver disease	6 (6)
Malignancy	16 (15)
Immunodeficiency^b^	9 (8)
Treatment^c^	
Oxygen supply, no. (%)	53 (49)
Non-invasive ventilation, no. (%)	2 (2)
Invasive ventilation, no. (%)	16 (15)

^a^The body-mass index is the weight kilograms divided by the square of the height in meters

^b^Due to disease or ongoing immunosuppressive treatment: renal transplantation (N = 1), psoriasis vulgaris (N = 1), Morbus Hashimoto (N = 1), leukaemia (N = 1), lymphoma (N = 3), gout (N = 1), polyarthritis (N = 1)

^c^All patients needing non-invasive or invasive ventilation were supplied with oxygen before ICU admission

### Iron deficiency and anemia

Two months after COVID-19 onset, 30% of all subjects still presented with ID. Of these, 13% had absolute ID and 17% functional ID according to TSAT and serum ferritin based definitions. Anemia was found in ten subjects (9.2%) and was more frequent in males (12%) than females (5%). Disease severity strongly correlated with the prevalence of anemia, as 90% of anemic patients previously had severe to critical COVID-19. Anemic patients primarily suffered from AI (70%) or combined forms of AI and IDA (20%), whereas IDA was only found in one patient. Notably, patients suffering from anemia demonstrated significantly higher IL6 (p = 0.009) and CRP (p = 0.031) concentrations as compared to non-anemic patients.

### Post-acute signs of hyperinflammation, coagulopathy and hyperferritinemia

**Fig. 1:**
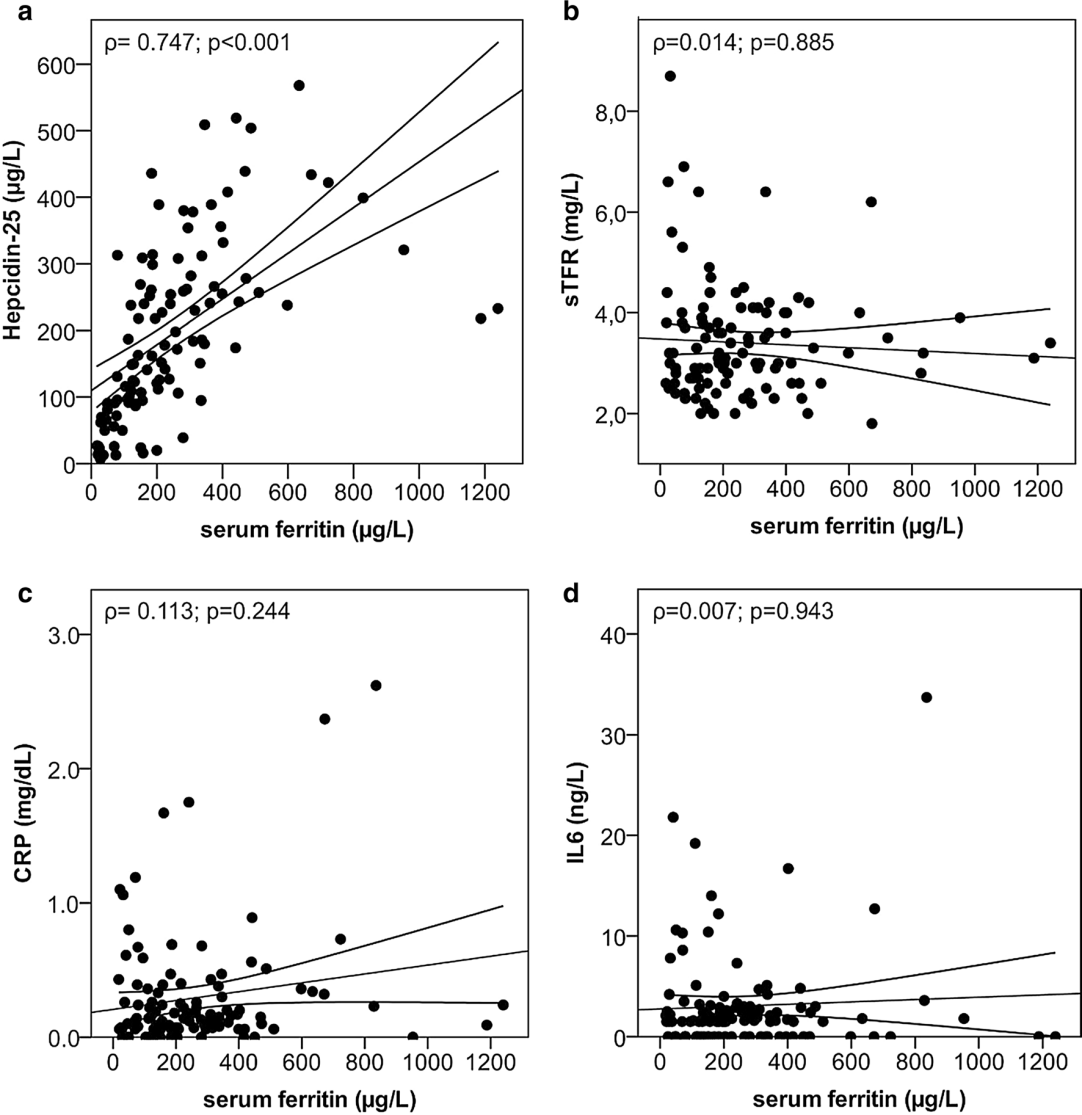
Serum markers of iron homeostasis in post-acute COVID-19 according to disease severity. Correlations of **a** hepcidin-25, **b** soluble transferrin receptor (sTFR), **c** C-reactive protein (CRP) and **d** interleukin-6 (IL6) with serum ferritin are shown. ρ indicates the correlation coefficient as calculated with Spearman-rank test

In the post-acute phase of COVID-19, a high proportion of individuals still presented with alterations of circulating biomarkers (Table 2). Most prominently, hyperferritinemia was still present in 38% of all subjects and was far more frequent in male (48%) as compared to female (23%) subjects (p = 0.009). Notably, serum ferritin strongly correlated with serum hepcidin concentrations, but not with markers of cellular iron demand (e.g. soluble transferrin receptor) or markers of inflammation such as CRP or IL6 (Fig. 1). Accordingly, serum hepcidin was positively correlated with TSAT (ρ = 0.328, p < 0.01), and negatively correlated with sTFRF index (ρ = − 0.439, p < 0.01), whereas markers of inflammation such as IL6 or CRP were not related to hepcidin levels. Of note, only a minor proportion of individuals presented with persisting mild elevations of inflammatory biomarkers. For instance, IL6 (cut-off > 7 ng/L) was increased in 12% and CRP (cut-off > 0.5 mg/dL) in 16% of the study participants, respectively.

**Table 2:** Serum biomarkers in post-acute COVID-19 according to disease severity

Disease severity	Mild (N = 22)	Moderate (N = 34)	Severe (N = 53)	p value
Markers of iron homeostasis				
Iron, µmol/L	18 ± 6	16 ± 6	15 ± 6	0.174
Transferrin saturation, %	27 ± 11	26 ± 9	24 ± 10	0.434
Ferritin, µg/L	139 ± 118	260 ± 183	317 ± 271	**0.001**
Soluble transferrin receptor, mg/L	2.9 ± 0.8	3.2 ± 0.9	3.8 ± 1.3	** < 0.001**
sTFRF index, (mg/L)/(µg/L)	1.5 ± 0.5	1.4 ± 0.5	1.7 ± 0.9	0.295
Hepcidin-25, µg/L	14 ± 10	22 ± 14	20 ± 13	0.073
Hematological parameters				
Hemoglobin, g/L	139 ± 12	138 ± 13	139 ± 17	0.650
Leucocytes, 10^9^ cells/L	5.7 ± 1.6	6.1 ± 2.2	6.4 ± 2.1	0.253
Thrombocytes, 10^9^ cells/L	251 ± 47	254 ± 60	259 ± 78	0.998
Markers of inflammation				
Interleukin 6, ng/L	1.45 ± 2.1	1.96 ± 1.9	4.43 ± 6.6	**0.017**
C-reactive protein, mg/dL	0.2 ± 0.3	0.2 ± 0.2	0.4 ± 0.6	0.067
Pro-calcitonin, µg/L	0.03 ± 0.03	0.03 ± 0.03	0.04 ± 0.04	0.332

Data are presented as mean ± 1 SD. Disease severity was categorized according to the need of medical treatment: mild, outward treatment, moderate, inward treatment, severe, inward treatment respiratory support (oxygen supply or mechanical ventilation). p values depict significant differences between severity groups

*sTFRF index* soluble transferrin receptor/log ferritin index

### Alterations of iron handling and immune effector function in peripheral blood mononuclear cells

**Fig. 2:**
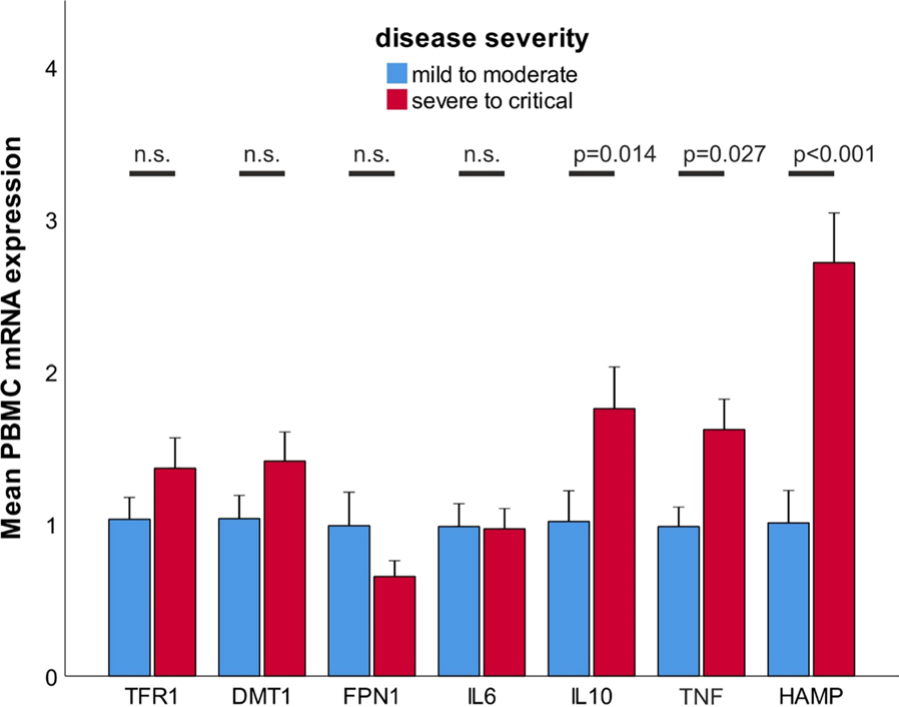
Post-acute mRNA expression of key modulators of iron homeostases and monocyte-derived cytokines in peripheral blood mononuclear cells of COVID-19 patients. Relative ΔΔCT mRNA expression as compared to levels in patients with mild to moderate COVID-19 are shown. Disease severity was categorized according to the need of medical treatment: mild to moderate, outward treatment or inward treatment without respiratory support; severe to critical, inward treatment with the need for respiratory support (oxygen supply or mechanical ventilation). p values depict significant differences between severity groups as calculated with Mann–Whitney *U* test, error bars indicate 1 standard error; N = 109. *TFR1* transferrin receptor 1, *DMT1* divalent metal transporter 1, *FPN1* ferroportin-1, *IL6* interleukin 6, *IL10* interleukin 10, *TNF* tumor necrosis factor, *HAMP* hepcidin antimicrobial peptide, *n.s.* not significant

To shed light on the regulation of iron metabolism and its impact on monocyte immune effector functions at the cellular level, we investigated the mRNA expression of key mediators of iron homeostasis as well as cytokine expression in PBMCs. In line with serum hepcidin measurements, we also found increased hepcidin mRNA (HAMP, for hepcidin antimicrobial peptide) expression in PBMCs isolated from subjects who previously had severe to critical COVID-19 as compared to those who suffered from milder disease (Fig. 2). Notably, mRNA levels of genes involved in cellular iron uptake, excretion and distribution, such as transferrin receptor 1 (TFR1), divalent metal transporter 1 (DMT1) and ferroportin-1 (FPN1), were not significantly associated with the severity of previous acute COVID-19. In contrast, the immune effector function of PBMCs was related to COVID-19 severity, as mononuclear cells obtained from patients, who suffered from severe to critical disease, demonstrated higher levels of interleukin 10 (IL10, p = 0.044) and tumour necrosis factor (TNF, p = 0.024) mRNA expression as compared to subjects with a milder course of COVID-19 (Fig. 2). Notably, PBMC mRNA expression of hepcidin was not related to monocyte cytokine expression, whereas H-ferritin mRNA concentrations of PBMCs correlated with TNF (ρ = 0.388, p < 0.001), IL10 (ρ = 0,399, p < 0.01) and lipocalin 2 (ρ = 323, p < 0.01) mRNA expression, but not with hepcidin (ρ = 0.609, p < 0.001).

### Association of hyperferritinemia with COVID-19 disease severity

**Fig. 3:**
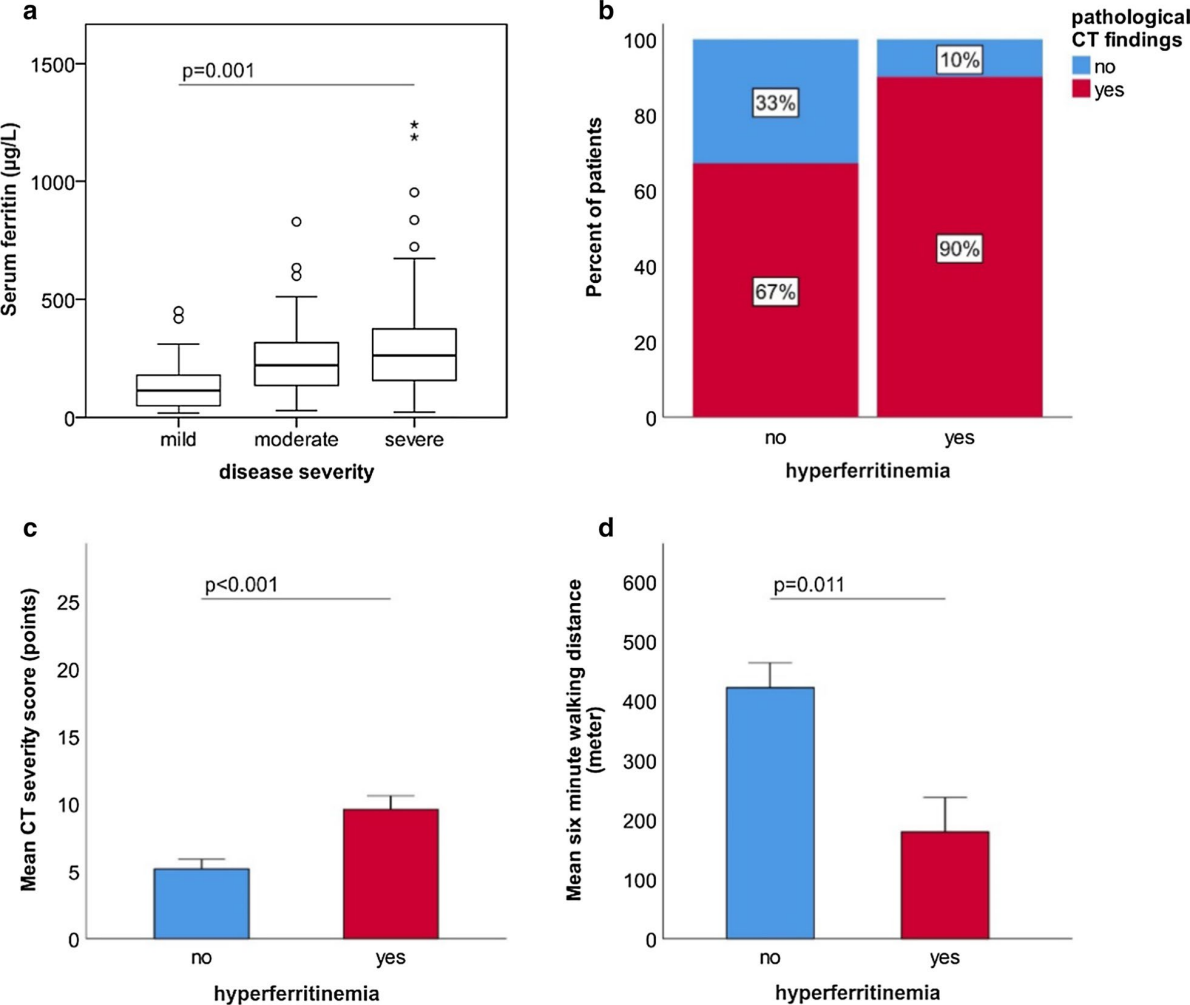
Association of post-acute hyperferritinemia with COVID-19 severity. **a** Serum concentrations of ferritin according to disease severity (mild: outward treatment; N = 22; moderate: inward treatment without respiratory support, N = 34; sever: inward treatment with additional respiratory support or intensive care unit admission, N = 53). **b** Frequency of lung pathologies detected with computed tomography (CT) scan 60 days after disease onset in patients with (N = 41) or without (N = 68) hyperferritinemia. **c** The severity of pathological CT findings according to the evaluation by two independent experts. The severity of lung involvement detected by CT was graded for each lung lobe and a sum score for the total lung was calculated (0–25 points). N = 109. **d** Six-minute walking distance in patients with (N = 12) or without (N = 11) hyperferritinemia. p values are reported according to the Kruskal–Wallis test (**a**) or the Mann–Whitney *U* test (**c**, **d**)

**Fig. 4:**
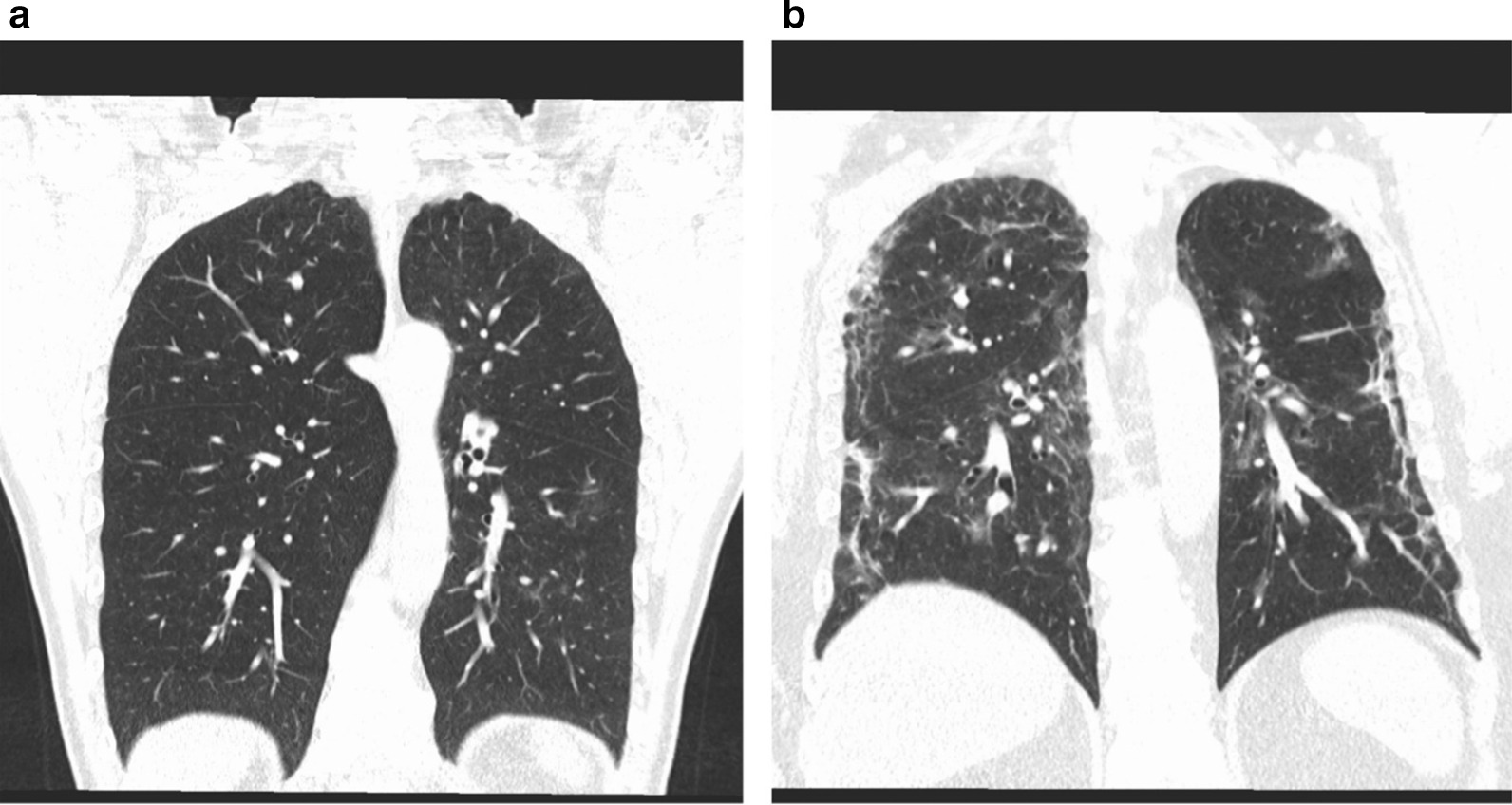
Representative CT scans of COVID-19 patients with or without hyperferritinemia. When comparing lung pathologies in CT scans 60 days after COVID-19 onset, patients with persisting hyperferritinemia presented with significantly more severe lung pathologies. A representative CT scan of two individuals without (**a**) and with (**b**) hyperferritinemia are shown

Strikingly, persisting elevations of serum ferritin levels were not only associated with alterations of PBMC cytokine expression but were related to the severity of COVID-19, as patients with a history of more severe disease demonstrated significantly higher serum ferritin concentrations as compared to individuals with milder disease (Fig. 3a). This finding was underlined by CT evaluation 60 days after disease onset, which revealed that in patients with persisting hyperferritinemia pathological CT findings were more frequent and more severe as compared to those with normal ferritin levels (Fig. 3b, c and Fig. 4). In line with this observation, in a subgroup of 23 study participants, who were evaluated with a six-minute walking test (SMWT), hyperferritinemia was associated with a decreased walking distance (Fig. 3d). Notably, in comparison to individuals with normal ferritin levels, patients with hyperferritinemia did not significantly differ in age, gender, frequency of co-morbidities or signs of inflammation, which would otherwise explain the difference in walking performance.

## Discussion

The rapidly emerging COVID-19 pandemic has overloaded the health care system in many countries worldwide, leaving few capacities to perform prospective trials for this new disease. Still, retrospective analyses have rapidly expanded our knowledge about COVID-19 and resulted in the discovery of a plethora of typical features of the disease [1, 27]. One of these features is the frequent emergence of disturbances of iron homeostasis in COVID-19, most prominently reflected by the high incidence of hyperferritinemia [1, 14, 28]. To date, it is still a matter of debate if disturbances of iron handling are just a reflection of the physiological adaption to the infectious disease or if dysregulated iron homeostasis contributes to COVID-19 pathobiology and disease outcome [15, 16]. The latter assumption is supported by the observation that hyperferritinemia is associated with increased mortality in COVID-19. Mechanistically, it has been suggested that hyperferritinemia and hepcidin dysregulation are related to iron toxicity and may contribute to end-organ damage in COVID-19 [1, 15, 28]. This theory is supported by previous data demonstrating that inflammation induces iron-dependant peroxidation processes resulting in cellular apoptosis, a process which is referred to as ferroptosis [29, 30]. Additionally, cellular iron overload is related to the production of reactive oxygen and nitrogen species, which may contribute to tissue damage [31]. Notably, iron dysmetabolism and ferroptosis have also been linked to typical COVID-19 associated symptoms, such as cognitive impairment and anosmia [15, 29]. In line with these observations, we herein describe persistent hyperferritinemia to be more frequent in subjects who suffered from severe or critical COVID-19 as compared to those who had milder disease. Importantly, patients with hyperferritinemia at follow up demonstrated a reduced performance status and persistent pathological finding in pulmonary CT scans.

It has been suggested that the COVID-19 related inflammation may be the main cause of COVID-19 associated iron disorders, as an inflammation-driven dysregulation of iron homeostasis is well established [4, 6, 7]. We herein demonstrate that especially severe COVID-19 causes prolonged alterations of iron handling even at a systemic level, as hyperferritinemia and increased expression of hepcidin are still found in a relevant proportion of patients two months after COVID-19 onset. Notably, our prospective analysis of post-acute COVID-19 associated iron dysmetabolism showed that hyperferritinemia was primarily related to systemic hepcidin expression, whereas no link to persisting inflammation could be established when studying circulating biomarkers. Thus, these data suggest, that in post-acute COVID-19, hepcidin expression is rather driven by iron levels than by persisting inflammatory processes. Of note, our data reveal that dysbalances of iron distribution, which emerge during acute COVID-19, result in prolonged disturbance of iron handling, which per se may impact on the resolution of inflammation and immune effector function of host immune cells. For instance, we previously reported that an acute increase of PBMC iron concentrations induces pro-inflammatory cytokine expression, such as TNF and IL6 production [18]. This mechanism may contribute to uncontrolled cytokine release, as found during the COVID-19 associated “cytokine storm”, as well [1]. In this context, we herein demonstrate that mononuclear cells, which were isolated from subjects following severe COVID-19, had higher mRNA expression levels of cytokines such as TNF and IL10, as compared to individuals with milder disease. Ferritin mRNA regulation at the cellular level was correlated with both cytokine expressions as well as markers of iron homeostasis. This is in line with data that TNF and IL-10 are strong inducers of ferritin expression and suggests that increased ferritin expression may reflect ongoing subclinical inflammation [32, 33]. The persistence of pathological radiological findings in CT and a reduced physical performance, as evident by reduced endurance in the SMWT, of patients with high ferritin levels would support this notion. Accordingly, ferritin may be directly involved in pathologic inflammation and lung injury as ferritin has been reported to act as a pro-inflammatory mediator [17].

In the context of COVID-19 related iron dyshomeostasis, monocytes and macrophages may play a pivotal role. Monocytes and macrophages are crucial mediators of inflammation and inflammation-driven iron sequestration, whereas their immune effector function is altered by iron availability [34]. Thus, these cells may be specifically exposed during COVID-19 and alterations of monocyte/macrophage iron handling may impact on the course of COVID-19 [8, 35].

Finally, we herein demonstrate that disturbances of iron homeostasis can persist for at least two months after the onset of COVID-19 and that prolonged hyperferritinemia is associated with persisting lung pathologies and a reduced physical performance status of COVID-19 patients. This would also suggest that determination of ferritin could be an easy accessible biomarker to monitor the persistence of pathologies following COVID-19. Whereas the herein presented data is observational, thus does not provide evidence for causality, these observations warrant further mechanistic evaluation and may significantly improve the understanding of COVID-19 pathobiology.

## Conclusion

In summary, we herein demonstrate that COVID-19 is associated with prolonged alterations of iron homeostasis, which per se are linked to a more severe initial disease but also persisting radiological pathologies in the lung and impaired physical performance of patients. Dysbalanced iron homeostasis is linked to tissue damage and impaired host-immune function, thus it is likely that iron disorders are not only an innocent bystander, but may significantly contribute to the course of COVID-19. Conclusively, further mechanistic evaluations of the role of iron homeostasis in COVID-19 are highly warranted.

## Supplementary information

**Additional file 1: Figure S1.** Enrolment of CovILD study participants.** Table S1.** List of primers and probes for RT-PCR analysis of PBMC mRNA expression patterns.

## Data Availability

All relevant data is included in the manuscript or Additional file 1.

## Abbreviations

AI: Anemia of inflammation
COVID-19: Coronavirus disease 2019
CRP: C-reactive protein
FPN1: Ferroportin 1
DMT1: Divalent metal transporter 1
GGO: Ground-glass opacities
Hb: Hemoglobin
IDA: Iron deficiency anemia
IL6: Interleukin 6
IL10: Interleukin 10
ILD: Interstitial lung disease
ID: Iron deficiency
PBMCs: Peripheral blood mononuclear cells
SARS-CoV-2: Severe acute respiratory syndrome coronavirus 2
SMWT: Six-minute walking test
sTFRF index: Soluble transferrin receptor/log ferritin index
TNF: Tumour necrosis factor
TFR1: Transferrin receptor 1
TSAT: Transferrin saturation
