# Supplementary material for: Persisting alterations of iron homeostasis in COVID-19 are associated with non-resolving lung pathologies and poor patients’ performance: a prospective observational cohort study
Source: Respir Res. 2020 Oct 21;21:276. doi: 10.1186/s12931-020-01546-2 (PMC7575703; doi:10.1186/s12931-020-01546-2)
Supplement: Supplementary file 1 — Additional file 1: Figure S1. Enrolment of CovILD study participants. Table S1. List of primers and probes for RT-PCR analysis of PBMC mRNA expression patterns. [file 12931_2020_1546_MOESM1_ESM.docx]

**Supplementary Material**

| 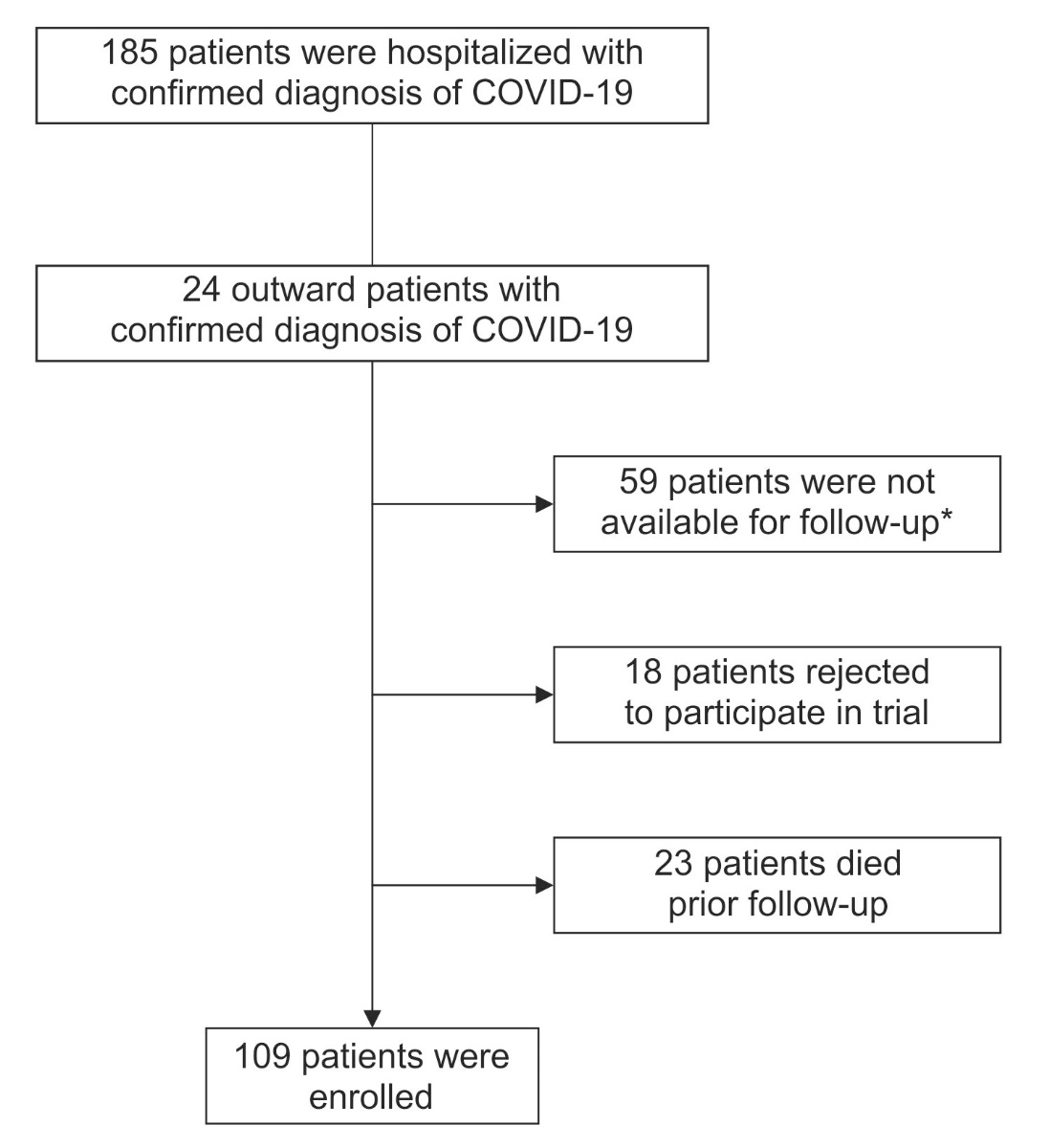 |
| --- |
| **Figure S1:** **Enrolment of CovILD study participants**  *non-available patients could not attend the study centre in Innsbruck because they left the country after hospital discharge or resided too far away from the study centre to attend regular follow-ups. |

| **Table S1 List of primers and probes for RT-PCR analysis of PBMC mRNA expression patterns** | | | | |
| --- | --- | --- | --- | --- |
| **TaqMan** | **forward primer** | **reverse primer** | | **probe** |
| β-TUBULIN | TCCTTCAACACCTTCTTCAGTGAGACG | GGTGCCAGTGCGAACTTCATCA | | ATGTGCCCCGGGCAGTGTTTGTAGACTTG |
| DMT1 | GTGGTCAGCGTGGCTTATCTG | CCACAGTCCAGGAAGGACATG | | TGTTCTACTTGGGTTGGCAATGTTTGATTGC |
| FPN1 | TGACCAGGGCGGGAGA | GAGGTCAGGTAGTCGGCCAA | | CACAACCGCCAGAGAGGATGCTGTG |
| HAMP | TTTCCCACAACAGACGGGAC | AGCTGGCCCTGGCTCC | | CAGAGCTGCAACCCCAGGACAGAGC |
| H-FERRITIN | AGAAAACTCAGCGACTGCCAG | TAGTTTTGGCGCACTTGCC | | ACCGCTGACCGCCCCGCT |
| TFR1 | TCCCAGCAGTTTCTTTCTGTTTT | CTCAATCAGTTCCTTATAGGTGTCCA | | CGAGGACACAGATTATCCTTATTTGGGTACCACC |
| **SYBR-Green** | **forward primer** | | **reverse primer** | |
| IL6 | CCACTCACCTCTTCAGAACG | | CATCTTTGGAAGGTTCAGGTTG | |
| IL10 | GGGAGAACCTGAAGACCCTCA | | TGCTCTTGTTTTCACAGGGAAG | |
| LCN2 | GGCTTCACAGAGGATACCATTGT | | AGCCCTGGGGAGTCCTATTG | |
| TNF | GGTGCTTGTTCCTCAGCCTC | | CAGGCAGAAGAGCGTGGTG | |
